# Children’s eating behaviours and related constructs: conceptual and theoretical foundations and their implications

**DOI:** 10.1186/s12966-023-01407-3

**Published:** 2023-02-15

**Authors:** Alan Russell, Elena Jansen, Alissa J. Burnett, Jookyeong Lee, Catherine G. Russell

**Affiliations:** 1 College of Education, Psychology and Social Work, Flinders University, Bedford Park, South Australia Australia; 2 Division of Child & Adolescent Psychiatry, Department of Psychiatry & Behavioral Sciences, Johns Hopkins University School of Medicine, Baltimore, USA; 3 Institute for Physical Activity and Nutrition (IPAN), School of Exercise and Nutrition Sciences, Deakin University, Geelong, Australia; 4 CASS Food Research Centre, School of Exercise and Nutrition Sciences, Deakin University, Geelong, Australia

**Keywords:** Child, Appetite, Eating behaviour, Survey methodology, Appetite self-regulation, Caloric compensation, Eating in the absence of hunger, Delay-of-gratification, Developmental trajectories, Fussiness

## Abstract

**Background:**

There is a substantial body of research on children’s eating behaviours (e.g., food responsiveness and fussiness) and related constructs (e.g., eating in the absence of hunger, appetite self-regulation). This research provides a foundation for understanding children’s dietary intakes and healthy eating behaviours, as well as efforts at intervention, whether in relation to food avoidance, overeating and/or trajectories to excess weight gain. The success of these efforts and their associated outcomes is dependent on the theoretical foundation and conceptual clarity of the behaviours and constructs. This, in turn contributes to the coherence and precision of the definitions and measurement of these behaviours and constructs. Limited clarity in these areas ultimately creates uncertainty around the interpretation of findings from research studies and intervention programs. At present there does not appear to be an overarching theoretical framework of children’s eating behaviours and associated constructs, or for separate domains of children’s eating behaviours/constructs. The main purpose of the present review was to examine the possible theoretical foundations of some of the main current questionnaire and behavioural measures of children’s eating behaviours and related constructs.

**Methods:**

We reviewed the literature on the most prominent measures of children’s eating behaviours for use with children aged ~ 0–12 years. We focused on the explanations and justifications for the original design of the measures and whether these included theoretical perspectives, as well as current theoretical interpretations (and difficulties) of the behaviours and constructs.

**Results:**

We found that the most commonly used measures had their foundations in relatively applied or practical concerns rather than theoretical perspectives.

**Conclusions:**

We concluded, consistent with Lumeng & Fisher (1), that although existing measures have served the field well, to advance the field as a science, and better contribute to knowledge development, increased attention should be directed to the conceptual and theoretical foundations of children’s eating behaviours and related constructs. Suggestions for future directions are outlined.

## Background

Children’s appetite, dietary intake and related phenomena, including eating behaviours, are profoundly relevant to the health and wellbeing of individuals, families, and societies. Presently, there are concerns about the state of children’s diets and weights in many countries [2–5], which are increasing children’s risk of acquiring chronic diseases later in life [4, 6]. Children’s diets and weight partly stem from children’s eating behaviours and related constructs (herein called “eating behaviours”). Indeed “eating behaviours” has emerged as one of the core components of research and understanding of children’s dietary food intake. A plethora of models and theories has emerged from a range of disciplinary perspectives that are directed to understanding elements and processes in relation to appetite, eating and their regulation. This has contributed to research on eating behaviours, especially in relation to the development of overweight/obesity [7–17]. These models and theories are relevant across the age-span, including for children. In parallel, efforts to measure children’s eating behaviours has continued apace. The measures have included questionnaires/self-reports as well as behavioural protocols, including laboratory-based observations. While the research on children’s eating behaviour is extensive, to advance knowledge and understanding, there is a need for more attention to be directed to the theoretical and conceptual foundations and associated definitions of measures [1].

Confusion or lack of clarity about the theoretical basis of children’s eating behaviours can contribute to poor construct definition. This, in turn can lead to difficulties in operationalisation and measurement of the eating behaviours and constructs, and therefore uncertainty in the interpretation of findings. The consequences could be many, including that research findings are less clear, the behaviour or construct of interest might not be measured, replication could be difficult and attempts to modify the target behaviour could fail. Almost 20 years ago, when discussing the field of children’s emotion regulation, Bridges et al. [18] argued that underlying the measurement of emotion regulation (implicit or explicitly stated) is a theoretical conceptualization of the physiological, behavioural and cognitive processes. They noted that there was a wide range of measures used and not enough emphasis on linking the measures with definitions of constructs and associated underlying processes. The domain of children’s eating behaviours currently seems to face some of the same issues. In particular, there seems to be limited identification or articulation of underlying theoretical frameworks.

What could be expected from a theoretical framework for children’s eating behaviours? It would probably begin with background assumptions or principles such as that children’s eating behaviours are partly biologically based, but subject to environmental influences and that the behaviours change or develop with age. A theoretical framework could provide for several overlapping domains or constructs of eating behaviours. Whether these domains or constructs are amenable to a single theoretical framework is a moot point. It is more likely there will be separate theoretical approaches to different eating behaviours and constructs. In each case, these approaches might involve separate concepts and definitions, with different assumptions and basic principles. Each would describe and explain different eating behaviour phenomena.

At present there does not appear to be either an overarching theoretical framework for children’s eating behaviour, or a set of well-articulated frameworks for each of the separate domains of eating behaviour. To help advance scholarship about the theoretical bases of children’s eating behaviours, the aims of the present review are to (a) highlight the importance and role of conceptual and theoretical foundations for children’s eating behaviour, (b) discuss the conceptual and theoretical foundations of some of the main current eating behaviours, and (c) discuss recent advances in theories and models of appetite, eating and their regulation and the associated implications for construct development, measurement and therefore understanding of children’s eating behaviour. The eating behaviours included as exemplars in the review are satiety responsiveness, food responsiveness/enjoyment of food, appetite self-regulation, food delay of gratification, eating in the absence of hunger, caloric compensation, food fussiness and food neophobia. Throughout, we were interested in processes in the development of the common questionnaire and behavioural measures, especially in relation to the role of theory in the formulation of constructs and associated measures, and then in whether and how these constructs are supported by recent theoretical models and related evidence. The review is partly informed by scholarship on the role of conceptual and theoretical foundations in social and developmental science.

## Methods

### Age range

The focus in the review is children aged ~ 0–12 years. These are ages during which research has examined the origins and development of eating behaviours and where there is a continuity in measures. This age period provides a substantial body of scholarship to address the three aims of the review.

### Defining “eating behaviours” and related constructs

There is ambiguity in both terminology and definitions about “eating behaviours” and related constructs in this age group. Further, there are several potentially relevant conceptual models, and many relevant constructs (c.f. the list of 33 appetite self-regulation-related constructs listed in [19]). Studies of children’s “eating behaviours” have considered what, how much, when, how, and why foods and beverages are consumed but they rarely provide a definition of “eating behaviours”. Children’s eating has typically been conceived in terms of individual appetitive traits such as food responsiveness, food fussiness or satiety responsiveness [20–23]. It is also possible to conceive of many of the eating behaviours or appetitive traits in terms of underlying processes. For example, food responsiveness could be seen as a process involving the perception of food cues together with the initiation of hedonic responses (liking, preference, appeal) and the engagement of liking or wanting and possibly inhibitory controls. In addition, eating behaviours have been conceived as skills, such as being able to inhibit responses to palatable food, to make decisions about portion sizes or to regulate eating rates. In addition, consistent with the trait-state distinction in relation to personality characteristics such as anxiety [24] or impulsivity [25, 26], child eating behaviours such as eating in the absence of hunger (EAH) or other behavioural measures have been considered to be more state-like [27, 28]. Finally, some constructs involve multiple components or processes. Examples include appetite self-regulation (ASR), EAH, caloric compensation, delay of gratification (DoG) and fussiness/food neophobia, as is evident from the multiple measures used in research on each of these constructs in the discussion below.

The literature we examined shows that “eating behaviours” are complex and multidimensional and have been variously defined. Generally, they are assumed to be constitutionally based, and therefore influenced by underlying biological processes, including genetic and epigenetic factors [11, 20, 29, 30]. However, they are also shaped by environmental factors [29], although this process could be bidirectional [10, 31]. Broadly, eating behaviours have been assumed to cover food selection, consumption and regulation of eating (what, when, why), hedonics, food reward value, food avoidance/approach, reactions to food cues, rate of eating, food and eating attitudes and beliefs/cognitions, habits. This broad understanding of eating behaviours informed our selection of constructs, theoretical models and measures to include in the review.

### Selection of children’s eating behaviours and constructs

For the body of the review, we chose eating behaviour measures and constructs that have been prominent in research in recent decades. We drew on integrative reviews of eating behaviours in recent decades (e.g., [27, 32–38] and scans of the research literature to assure that the most prominent measures were included. The selection was not intended to be exhaustive, rather to enable a discussion of prominent and representative measures. The selection involved questionnaire measures such as the Children’s Eating Behaviour Questionnaire (CEBQ) and their associated constructs, and behavioural measures such as EAH, caloric compensation, DoG and measures of food fussiness. We first searched for theoretical and conceptual foundations in the rationale and explanations for the questionnaires and behavioural measures in the original or early publications about the measures. Second, we searched more recent publications that have used the measures for comments and suggestions about theoretical foundations, including via interpretations of findings.

## Results

### Conceptual and theoretical foundations of behavioural constructs

In this section, we first draw on scholarship in psychological and developmental science about the importance of the conceptual and theoretical foundations of behavioural constructs, where it has been argued that these constructs should be rooted in a coherent theoretical perspective [18, 39, 40]. This could be by first theoretically deriving dimensions of the construct, as Rothbart et al. did in the case of child temperament [41]. The associated argument is that behaviours to be measured should be clearly defined, their theoretical underpinnings and scope articulated, and a description provided of how they have been operationalised. Eating behaviours are theoretical constructs devised to facilitate the description and analysis of behaviour associated with diet and eating. In this sense, they parallel other constructs in psychology and child development, such as temperament, emotion regulation, and social withdrawal. The meaning and measurement of these constructs has been enhanced by efforts to provide an account of their theoretical foundations [39, 41, 42].

With respect to questions about the role of theoretical foundations, child temperament is an instructive source of comparison with children’s eating behaviour. The conceptualisation of child temperament has a history of several decades, during which prominent proponents of different conceptualisations and associated methods and measures of child temperament emerged. Traditionally there are four main theoretical approaches to temperament (including Goldsmith, Buss & Plomin, Rothbart and Thomas & Chess) (see the roundtable discussion chaired by Goldsmith in 1987 [43] for an introduction as well as the chapter by Shiner and DeYoung [44]). In addition, Kagan and colleagues [45, 46] focused on temperamentally inhibited or uninhibited children.

Each of these theorists and approaches to temperament articulated a theoretical basis as the foundation of construct definition and measurement. In turn, each of the theoretical approaches is associated with questionnaire tools and laboratory-based or behavioural strategies for the measurement of the articulated theoretical constructs, and these have continued to be developed and refined. This has engendered research to compare the convergent and discriminant validity of different temperament questionnaires for young children to identify commonalities and conceptual boundaries [47]. A productive aspect of the richness of theoretical models and measurement strategies in temperament is that it has generated efforts to identify and agree about core dimensions of temperament [48, 49]. Individual scholars face a similar task of identifying significant dimensions of temperament when conceptualizing and designing research, such as the case of food neophobia where negative emotionality (fearfulness or withdrawal) has been determined as the aspect of temperament most relevant [50].

Scholarship on temperament has benefited from the attention to theoretical models and foundations in at least three ways: (1) questions about the construct and its dimensions have been enriched, enhanced, and enabled by the theoretical underpinning (as evident in the roundtable discussion, for example), (2) the development and evaluation of measurement instruments and procedures have been assisted by the theoretical context provided, and (3) the theoretical models have contributed to the interpretation and application of research results, including in areas such as children’s food neophobia [50]. Similar comments could be made about the benefits for scholarship of theoretical models in the field of self-regulation, where there has been a proliferation of models [51].

In contrast to this approach in the field of child temperament, as we outline below, child “eating behaviours” and related constructs seem generally to have origins in research about outcomes such as healthy diets or weight gain, obesity, or food avoidance. Often, as we argue below, the articulation of underpinning theoretical foundations has not appeared to be a priority: rather, the potential practical or applied significance of the behaviour seems to have been paramount. However, we argue that now is the time for greater consideration to be given to the underlying theoretical foundations of these constructs for both practical and theoretical benefits.

Recent discussions of social and emotional development have highlighted the importance of theoretical and conceptual foundations for measurement. Darling-Churchill and Lippman [52], for example, underscore the importance of conceptualization in order to identify the constructs in relation to social and emotional development. Central to the advancement of measurement in their view was clarity about the subdomains of social and emotional development and their associated measures. Comparable comments about measurement in other areas of psychology were made by Flake and Fried [40] who argue that even after decades of research and thousands of studies on depression, concerns remain about depression as a construct and its associated measures, and that this has limited advancements in the field. There seem to be similar issues in relation to children’s eating behaviours and related constructs where numerous subdomains of eating behaviours have been proposed, many without an articulation of their theoretical or conceptual origins and this limits both measurement and interpretation.

#### Meta-theories and theoretical frameworks

In addition to underlying theoretical perspectives, it is also helpful if constructs and measurement are linked and informed by broad theoretical frameworks [53] or meta-theories [54, 55]. There seems to be some consensus in developmental psychology around overlapping guiding frameworks variously labelled as a transactional model, a biopsychosocial approach, or a bioecological model [11]. We [10, 11] and others [56–58] have suggested that in relation to the development of children’s eating behaviour and obesity, a biopsychosocial approach (i.e., involving the interconnection of biological, psychological and socio-environmental factors) could also provide a useful general theoretical framework to guide the articulation of definitions, generate predictions, help in the design of measurements and assist in the unification of approaches and results. As outlined above when discussing definitions of children’s eating behaviour and related constructs, there is broad agreement, but not to a degree that could be described as a coherent or overarching theoretical framework. We suggest below that it might be necessary for separate frameworks for different domains of children’s eating behaviours, such as food approach behaviours, food fussiness, and appetite self-regulation.

#### Boundary problems in construct definitions

Boundary problems have been identified in psychological and developmental science as contributing to difficulties in construct definition and measurement. For example, problems in construct definition in relation to social and emotional development have included the “jingle and jangle fallacies” [59]. The jingle fallacy refers to the use of a single term to refer to a number of constructs while the use of different terms to refer to the same construct is the jangle fallacy. The jingle and jangle fallacies exist at least partly due to inadequacies in identifying boundaries between constructs and building measurement tools on inadequate theoretical foundations. In relation to construct boundaries, there is also the likely problem of what Darling-Churchill & Lippman [52] referred to as conceptual clutter in their discussion of early social-emotional development. Clutter arises from the lack of agreement about the definition of separate domains or dimension of social-emotional development, or in our case children’s eating behaviour. As we argue below, in the case of children’s eating behaviour, it is possible that constructs such as “food responsiveness”, “satiety responsiveness”, “eating in the absence of hunger”, “caloric compensation”, and “delay of gratification” are examples of the jingle fallacy as well as conceptual clutter.

### Conceptual and theoretical foundations of selected eating behaviours and related constructs

In this section we discuss conceptual and theoretical foundations of a selection of prominent child eating behaviours and constructs. We begin with the more commonly used questionnaire/parent report measures and their associated eating behaviours together with the specific traits of satiety responsiveness and food responsiveness. The construct of appetite self-regulation is then examined, followed by the behavioural protocols of eating in the absence of hunger, food delay of gratification and caloric compensation. Finally, we discuss food neophobia/food fussiness.

#### Questionnaire/parent-report measures and their eating behaviours

The CEBQ [60] and the parallel eating Baby Eating Behaviour Questionnaire (BEBQ) [61] have gained wide acceptance in research on children’s eating behaviour. Other relevant questionnaires include the Children's Dutch Eating Behaviour Questionnaire (DEBQ-C) [62, 63], the child and adolescent version of the Three-Factor Eating Questionnaire (CTFEQr17) [64] and the Child Self-Regulation in Eating Questionnaire [65].

The CEBQ and BEBQ were founded on ideas about genetic influences on obesity occurring through eating behaviours [66]. In constructing the questionnaire, Wardle and colleagues [60] selected six eating style constructs that they identified from behavioural and questionnaire research as contributing to overweight/obesity, as well as interviews with parents and extrapolation from adult theory and evidence on eating. Items were generated to assess the six constructs derived from the literature that they labelled as food responsiveness, external eating, appetite/enjoyment of food, satiety sensitivity, slowness in eating, fussiness and emotional overeating together with four additional constructs identified from the parent interviews, namely emotional undereating, appetite for drinks, social eating and distractibility. Items were culled after analysis of responses from three samples of families with young children. Factor analyses were used to confirm the empirical coherence of the individual subscales and structure of the overall questionnaire. Three points about the development of the CEBQ are relevant to the present discussion: (a) the constructs were primarily empirically derived, (b) the designers provided limited discussion or justification for the labels they used for each of the constructs, and (c) the constructs have subsequently been almost reified, with limited interrogation against recent theoretical developments. We argue, however, that a critical examination of the constructs is necessary to advance scholarship on children’s eating behaviours**.** Below we examine the constructs of food responsiveness and satiety responsiveness in relation to more recent theory and evidence.

The development of the BEBQ was described by the authors as being designed to measure four appetitive traits “that are thought to be important for weight” (61 p. 389). The constructs and items were based on the scales from the CEBQ, the literature on milk-feeding, and interviews with mothers. The Child Self-Regulation in Eating Questionnaire was developed from a previous review of the literature on child feeding and weight status and from qualitative data from a pilot study [65]. The development of the CTFEQr17 drew on the adult version of the Three-Factor Eating Questionnaire and used interviews with children and adolescents to gauge their understanding of the adult items and to create appropriate items accordingly. The original adult version was developed by selecting items from two existing questionnaires about restraint and latent obesity plus new items based on clinical experience. These questionnaires, then, appear not to stem in the first instance from theoretical conceptions of eating behaviours.

Results from subsequent studies utilising the CEBQ and BEBQ suggest that the constructs measured with these tools could suffer from limited construct definition, arising from overlap among the subscales. For example, the satiety responsiveness and slowness in eating subscales tend to correlate strongly, as do the food responsiveness and enjoyment of food subscales, although there is some variation across studies [67, 68]. Testing in different populations has at times largely confirmed the factor structure of the original CEBQ [69], but studies with diverse samples from a range of countries [70–77] have revealed poorer fits or variations from the original factor structure, suggesting that they may not reflect common underlying processes or that there are theoretical weaknesses in the conceptualisation of the constructs. Oyama et al. [71] could not replicate the factor structure of the CEBQ or BEBQ in a Samoan sample, and speculated that the wording of the questions could at least partly explain cross-cultural differences, but also that the appetitive traits measured by the CEBQ may suffer from being theoretically indistinct. It is also possible that there are cultural or individual differences in word meanings or images generated by the questions, a potential difficulty associated with questionnaires [78].

The DEBQ [63] was reportedly based on “psychosomatic theory”, which was related to emotional eating, “externality theory”, which was related to external eating (eating in response to food-related stimuli) and the theory of restrained eating that implicates loss of contact with internal signals of hunger and satiety. All three theories were selected because of their relevance to the development and maintenance of obesity. The authors selected items from three existing questionnaires to measure three constructs: restrained, emotional and external eating. Factor analysis over several studies was used to refine the items for each construct. In this case, the DEBQ appeared to draw on an underlying theoretical framework.

#### Satiety responsiveness

Most of the CEBQ or BEBQ subscales have individually been the subject of research, with the satiety responsiveness subscale being an important focus [79–81]. In the case of the CEBQ and BEBQ, the theoretical foundation of the satiety responsiveness subscale is somewhat unclear. The CEBQ subscale combines items about both satiation (e.g., my child leaves food on his/her plate at the end of a meal, my child gets full up easily) and satiety (e.g., my child cannot eat a meal if he/she has had a snack just before, and my child has a big appetite). Satiation has been defined in terms of the processes that bring a meal to an end and satiety as involving postingestive process that inhibit further eating (e.g., via feelings of fullness and suppression of hunger) and impacts the frequency of eating [82]. The fact that this CEBQ/BEBQ subscale is a component of “food avoidance” and is correlated with subscales such as food fussiness and slowness in eating [34, 60, 83], further complicates efforts at conceptual clarity and suggests that the jingle fallacy could be relevant here.

#### Food responsiveness/enjoyment of food

Articulating the conceptual boundaries and theoretical distinctness between other constructs in the CEBQ and BEBQ is also difficult, especially in the correlated subscales of “food responsiveness” and “enjoyment of food”. On inspection, the items in these subscales appear to overlap and are open to questions related to the jingle and jangle fallacies as well as being subject to a level of conceptual clutter. For example, items for the food responsiveness subscale include “my child’s always asking for food” and “given the choice, my child would eat most of the time”, while items for the enjoyment of food subscale include “my child enjoys eating” and “my child loves food”. As noted earlier, there does not seem to have been an explanation for the terms chosen as labels for these constructs. Theoretically and conceptually, it would help if there was an account of the separation and overlap of these subscales with other related constructs such as hedonic hunger/hedonic eating (eating for pleasure), food reward, food reward sensitivity, the power of food, the reinforcing value of food and food cue responsiveness.

#### Appetite self-regulation

Appetite self-regulation (ASR) has been identified as a core construct in relation to children’s weight gain and obesity [13, 27, 37, 84, 85]. ASR has been argued as pertaining to responding to hunger, satiation, and satiety cues [13, 86]. These processes are easy to define in a general way, but difficult to measure [87]. Further, they are implicated in ASR, but are not themselves ASR [37]. Scholarship is at present engaged in efforts to clarify the construct and its measurement [37]. The diversity of approaches is illustrated by the fact that Frankel et al. [88] defined and measured ASR by the “satiety responsiveness”, “enjoyment of food” and “food responsiveness” subscales of the CEBQ. In contrast, Monnert-Patris et al. [89] developed a parent-report questionnaire drawing on the concepts of “eating in the absence of hunger” and “caloric compensation”. Tan & Hollub [65] also developed a parent-report measure of ASR. It contained a disparate collection of eight items covering different presumed elements of ASR that were selected based on earlier research on parent feeding attitudes and practices [90]. In relation to theoretical and conceptual underpinnings, none of these scales appears to have been based on an articulated or agreed conceptualisation of ASR.

In recent years, there have been several new approaches to the conceptualisation of ASR. This includes the bottom-up, top-down model [37, 91, 92] also described as a dual processing model [93] and a similar model described by Reigh et al. [94]. In these models, ASR is conceptualised as involving bottom-up approach or avoidance reactions together with top-down regulatory control. Attention has been directed to conceptualising and measuring both the bottom-up processes, including hedonic responses to food and food cues [95–97], aversive or avoidant reactions to food as in food fussiness and food neophobia [36, 98], and the top-down regulatory processes. In the latter case, there have been investigations of the role of inhibitory control [99–102].

The complexity of the conceptualisation and measurement of ASR is illustrated by continuing debates and the use of a diversity of measures of inhibitory control and impulsivity [25, 103–108], where it is acknowledged that they themselves are multidimensional constructs. Bennett et al. [109] argued from their results that relationships between impulsivity and eating behaviour could be measure- and respondent- dependent. In addition to inhibitory control and impulsivity, it is instructive to consider the relevance of the theoretical foundations of general self-regulation to ASR. For example, Gagne et al. [110] outline a model with self-regulation and emotion-related self-regulation having foundations in Effortful Control (EC) and Executive Function (EF). There is a body of scholarship that attempts to locate aspects of the theoretical foundations of ASR in EC and EF [19, 58, 99, 111]. In this way, the conceptualisation and measurement of ASR can be informed by established domain-general models of self-regulation and other fundamental processes in child development.

#### Food Delay of Gratification (DoG)

Food delay of gratification is now a widely used measure in research on ASR [19, 112–114]. The most frequently used measure is a choice delay task, which emerged from the research of Mischel and colleagues on self-control in the 1950s and 1960s [115–117]. Here a child is offered a choice between an immediate but smaller food reward and a delayed but larger food reward [117]. Marshmallows were frequently used as the food reward, but a variety of other palatable foods has been used. An alternative is the sustained delay task [118, 119] in which the child makes an implicit choice and must sustain the choice to receive the reward later. The origins of the choice DoG task were in efforts by Mischel and colleagues to develop a laboratory assessment of children’s self-control. The traditional assumption was that a tendency to delay reflects increased self-control [118].

Over subsequent decades, increased attention has been directed at theoretical underpinnings of the task, to the interpretation/significance of children’s performance and to the possible long-term significance for child development. This scholarship has yielded several theoretical possibilities and insights that raise questions about what the DoG tasks actually measure. In early research, Mischel and colleagues [116] examined cognitive and attentive mechanisms in DoG performance and other covariates. Watts and colleagues [120, 121] more recently questioned whether DoG performance is a measure of self-control and about the contribution of cognitive ability. They discuss whether DoG can be conceived as a unique construct. Giuliani & Kelly [112] investigated relationships between performance on several cognitive tasks and DoG. In this case, these relationships were stronger for a tongue delay task in which the snack was placed on the child’s tongue, and the child needed to wait for a signal to eat it versus the traditional choice delay procedure. Duran & Grissmer [118] examined relationships between delay and measures of EF. Contrary to expectations, immediate gratification was related to better EF and to better school-related outcomes. They argued that immediate gratification could be adaptive among some children in some contexts (the children in their sample were mostly Black and from low-income families). Watts et al. [120] identify additional complexities associated with processes linking DoG to later outcomes.

There seem to be three common and divergent theoretical interpretations of DoG performance: (a) delay for the larger reward arises from increased sensitivity to the reward value of food, (b) choice for the immediate reward arises from greater impulsivity, and (c) delay for the larger reward is due to better top-down regulatory control [37]. Suor et al. [122] have also argued that there is a lack of understanding of processes contributing to children’s delay abilities.

There are two further possible complications about theoretical interpretations of DoG performance. The first is that there could be differences in underlying processes and outcomes associated with food versus non-food DoG tasks [19]. A second complexity has been expressed by Hughes et al. [86]. They argued that food DoG is not a measure of ASR, satiation, satiety, or energy balance regulation, and therefore is not a measure of ASR. Overall, from the early origins of the DoG task with a relatively practical purpose, variations of the measure have emerged, and the field has spawned a rich field of scholarship about possible theoretical underpinnings. However, if food DoG is included under the umbrella of eating behaviour, there is still a need for advances in theoretical aspects of the measure. This could be elucidated somewhat by attention to the abilities or factors that underpin performance, processes that are involved in the task itself, and processes associated with linkages between DoG performance and developmental outcomes.

#### Eating in the absence of hunger

The early development and use of the EAH protocol is especially associated with the work of Fisher and Birch [123–126] where the emphasis was on links between parents’ restrictive access to palatable foods and children’s consumption of those foods. The EAH protocol was used as a method of assessing this association. A theoretical rationale at the time was that restrictive/controlling feeding practices limit children’s opportunities to exercise/learn self-control and that these practices thereby impede the development of ASR. Another suggestion at the time was that EAH could be indicative of children’s responsiveness to environmental cues taking precedence over children’s own fullness. From an original purpose of investigating the possible effects of restrictive feeding on the consumption of palatable food, in the subsequent two decades, the EAH protocol has evolved to the point of being claimed as representing “objectively measured appetite regulation” [127]. But there is presently no agreed definition or conceptualisation of appetite regulation or ASR to support a claim such as this.

Indeed, the “eating in the absence of hunger” (EAH) construct and measures are also examples where there appear to be limitations in theoretical and conceptual foundations. In recent years, the EAH protocol has been conceptualised variously as measuring “satiety responsiveness”, “disinhibited eating”, “satiety”, “satiation”, as well as “food cue responsivity”, “hedonic eating”, along with a combination of two (“satiation” and “food cue responsivity”). Possible links with constructs such as the “relative reinforcing value of food” are also relevant [128]. While EAH is usually assumed to be about eating beyond satiation [35, 129], other researchers have conceptualised it as being about having eaten to satiety [130, 131]. Francis et al. [132] argued that EAH involves over-riding satiation and satiety cues. There seems to be some uncertainty amongst researchers about the role of satiation and satiety in EAH. Further, it is evident that EAH is not itself a measure of either satiation or satiety. EAH seems to reflect aspects of poor ASR and is possibly related to disinhibited eating [80, 126, 133]. It could be that children who do not EAH are more sensitive to satiation cues and/or less responsive to food cues. Taken together, these points suggest that the conceptualisation of the EAH construct would benefit from further clarification.

In relation to the validity of the EAH protocol, there is some evidence of associations between the CEBQ scales and results from the EAH protocol [134]. More usual, however, is for there to be limited associations between the EAH protocol and questionnaire and self-report measures of eating behaviour (e.g., from the CEBQ) or ASR. These results prompt questions about the conceptual foundations of the EAH protocol and interpretations of results [27, 28, 135–137]. Furthermore, while there is agreement about the general procedures for the EAH protocol, there are variations across publications in the details. The possible implications of these variations for the measurement and interpretation of EAH is unclear. For example, there are variations in relation to the content of the prior meal, the time between the meal and subsequent snack provided and in the specific components of the snack, and the offered alternative (e.g., toys, activities, books) [37]. Madowitz et al. [137] suggested that more research is needed to determine which aspects of EAH are being measured via questionnaires and the EAH protocol, implying that the two procedures may be measuring different constructs. With regard to relationships to diet and health outcomes, there is reasonably consistent evidence about the predictive validity of measures from the EAH protocol with respect to relationships with Body Mass Index (BMI) or overweight/obesity and increased energy intake [138–140].

It is apparent, then, that EAH is a multi-dimensional set of behaviours and processes with several possible theoretical foundations. However, there is no coherent agreement about it conceptually or what it measures. Differences between questionnaire/report and behavioural measures add to uncertainty about the construct. It is unclear whether EAH could reflect higher levels of the hedonic value of food (the appeal, preference, or reward value of the food) or poorer capacities for inhibitory control [141, 142] or some combination of these. Overall, EAH is a widely accepted and used protocol with evidence of links to children’s energy intakes, weight gain, and BMI. Nevertheless, it is also a protocol that is in search of a theoretical foundation. Until there is more conceptual clarity, including about construct boundaries and underlying mechanisms, there will remain some confusion about what the protocol measures, how to interpret results, and how to position it in relation to other constructs such as ASR.

#### Caloric compensation

The term “caloric compensation” is used for a collection of protocols that developed or emerged from early efforts to provide children with a defined preload, with subsequent intake measured to determine whether or to what extent the subsequent intake compensated for the preload [143]. This was measured in terms of whether or how much the intake was adjusted or increased in response to the caloric content of the preload [143]. Since its inception, there have been multiple variations on the “caloric compensation” protocol, aligned to a variety of purposes. These purposes include whether or how the child adjusts to portion size, to a prior meal, from day-to-day, and according to the characteristics of the preload (e.g., nutrient content, energy density, and food form) [94, 144–149]. This research also shows that there have been differences in the time delay between preload and meal, and the number and characteristics of the food provided (preload and subsequent meal) including sensory qualities and palatability. In short, there is no standardised procedure or protocol. This adds to complexities associated with the conceptual or theoretical underpinning of the construct.

Underlying the set of compensation procedures are questions about what factors influence children’s ability to sense and respond to hunger and satiety cues, whether children can “perceive” calories [143] and, what the mechanisms are that are involved in compensation. It is generally assumed that ASR is somehow a component of compensation, however, it is evident that compensation does not directly measure ASR, nor is it a measure of satiety [37] although it could help in identifying general phenotypes such as low or high satiety phenotypes [150]. Presently, researchers appear to be confident about being able to measure compensation and relate this to prior intake, yet the reasons for the level of compensation and factors contributing to the compensation are less clear.

An early impetus for the caloric compensation protocol appears to have been questions about children’s abilities to adjust their intake based on caloric density, and possible implications of this ability for weight gain/adiposity [143]. Gradually, theoretical underpinnings are emerging, often tied either to characteristics of the prior intake (e.g., energy density or food form) or to possible compensation mechanisms or processes. As noted above, discrepant outcomes between different measures of eating behaviour constructs such as self-report versus observational measures continue to raise questions not only about construct validity, but also whether different measures capture different constructs. Similar questions could be raised about variations in measurement protocols for caloric compensation with implications for construct definition and measurement.

#### Fussiness and food neophobia

When Lumeng and Fisher [1] called for researchers to address the conceptualization of eating behaviours, they cited “fussiness” as one example where there is confusion in construct definitions and measurement. They noted that “food fussiness”, “food neophobia”, “food selectivity”, and “food rejection” are all related or overlapping concepts. Indeed, “food fussiness” is a term that appears to suffer from the jangle fallacy. A widely accepted definition of “food fussiness” is avoidance of both new and familiar foods [151], with “food neophobia” (rejection of new foods) a subcomponent of “food fussiness” [98, 152]. There are many terms that refer to the concept of “food fussiness” including “food pickiness”, “food avoidance”, “food refusal/rejection”, “choosy eating”, “selective eating”, “faddy eating”, and “finicky eating”, and a number of associated definitions [36, 38, 153–155]. There is also a variety of assessment tools used to measure these concepts ranging from single item parent reported perceptions to observational measures and validated questionnaires, yet the theoretical foundations of the various measures are rarely articulated.

Dovey et al. [152] highlighted the need for consistent measurement of “food fussiness”, noting that the use of measures with poor reliability and validity would lead to “further confusion and problematic theoretical interpretation” (p. 188). There remain challenges associated with measuring “food fussiness” arising from the diversity of measures [156]. Rejection of *new* foods is considered as an evolutionarily natural behaviour for a food safety purpose to avoid the toxic and harmful [151], however, the rejection of *familiar* foods does not have a clear underlying process or theoretical foundation as a component of “food fussiness” [155]. While the two constructs are theoretically distinct and are predicted by different factors, they are also often highly correlated [152], a challenge Rioux and colleagues recognised and attempted to address with the development of a “food rejection scale” [157]. A broad theoretical framework that acknowledges biological, psychological and social factors in food liking and preferences could be helpful in conceptualising and measuring fussy eating and associated constructs.

## Discussion

Since the development of the measures of children’s eating behaviour and related constructs discussed here, contributions to research and theory have progressed; especially in relation to the development of theoretical models of eating behaviour that can contribute to the conceptualization and measurement of children’s eating behaviour and related constructs. Here we discuss how three of these models could help.

One theoretical advancement has been in the development of biopsychosocial models of children’s eating behaviours. For example, Chawner & Hetherington [57] outline an integrated (biopsychosocial) model for the behaviour of liking and consuming vegetables. Anzman-Frasca et al. [158] adapted Gottlieb’s [159] theory of probabilistic epigenesis (also a biopsychosocial model) to children’s food preference and behaviour. Both models highlight the need to focus on definitions and measurement of liking and food preferences aspects of children’s eating behaviour with biopsychosocial and psychophysiological dimensions of these behaviours providing the theoretical foundations. Biopsychosocial models highlight additional components of children’s eating behaviours. For example, visual, tactile and flavour exposure, behavioural and biological responses to visual, tactile and flavour experiences, positive and negative food experiences, and willingness to taste. Also, cultural contexts and practices in children’s food and eating have been identified in biopsychosocial models. These models assist in the conceptualisation of eating behaviours, for example, as states and/or traits, as developmental and as context-dependent behaviours.

A second area relates to several recent theoretical developments and associated models that derive from neuropsychological and health neuroscience perspectives, which appear to make helpful contributions to the conceptualisation and measurement of children’s eating behaviours. The theoretical developments from this perspective have come in several areas, including homeostasis, hedonics and food reward, ASR and inhibitory control, and satiety. This work integrates psychological aspects of eating behaviour and neurological/biological processes. The presentation of a health neuroscience perspective and model set out by Lowe et al. [160, 161] illustrates the advances in this area and how this theoretical perspective can inform the conceptualisation and measurement of children’s eating behaviour. They draw on Erikson et al. [162] who describe the health neuroscience perspective as one in which the brain influences, and is influenced by, physical health. In the Lowe et al. model, physical health is especially related to consumption of calorie-dense food and obesity. Lowe et al. [161] point out that there has been considerable focus on the reward system and heightened responsivity to food cues in neurobehavioural models. They suggest that emphasis also needs to be placed on prefrontal cortex structure and functionality and associated consequences for executive functions (EF), especially inhibitory control.

There is now a collection of research, models and theoretical developments from a neuroscience and neuropsychology perspective in relation to children’s eating behaviour. These include theory and research about the hedonic system, the neurobiology of food reward circuitry, cognitive control circuitry and associated attention to inhibitory control [95, 96, 101, 163–167]. The hedonic component of eating behaviour has also been separated into the individual elements of liking and wanting [168–170]. There has been considerable attention to the role of inhibitory control, EF and impulsivity in children’s eating behaviour together with associated neural mechanisms [105, 132, 142, 169, 171–178]. Theoretical models such as proposed by Lowe et al. [161] and the associated literature suggests a framework of children’s eating behaviour and related constructs that would include: consumption of calorie-dense food, behavioural and inhibitory control, reward sensitivity, EF (including EF impairments), food cue sensitivity, food habituation, evaluation of food nutritional value, impulsivity and food decision-making processes. An implication is that measurement would include biological, psychophysiological and behavioural methods.

A third relevant theoretical framework that could inform the conceptualisation of children’s eating behaviours is the Satiety Cascade, which was proposed in 1987 by Blundell and colleagues [179]. It is described as a psychophysiological framework that combines physiological, behavioural and psychological processes [82, 166, 180–182]. The Satiety Cascade is a theoretical framework that involves sensory and cognitive processes in the pre-ingestion phase, satiation associated with the post-ingestion phase and satiety associated with the post-absorptive phase. It links eating motivations and behaviours to cognitive and physiological processes across the three phases. There is an extensive literature on biomarkers of satiation and satiety [183] and an associated literature on the assessment of satiation and satiety using biological and behavioural measures [82, 150, 166, 180, 181, 184, 185] as well as brain imaging following exposure to food-related cues [169].

Overall, it is apparent that the evidence together with the theoretical models accumulating from biopsychosocial, neuroscience and neuropsychology perspectives plus the satiety cascade provide a foundation for advances in the conceptualization and measurement of eating behaviour in children. The focus of this evidence and theoretical models seems to have been in relation to (a) hedonics and food reward, (b) inhibitory control or regulatory processes, (c) impulsivity and (d) satiation and satiety. There is now scope for advances in construct definition and measurement strategies in each of these areas that include self-report/questionnaire, behavioural, psychophysiological, and neural measures of children’s eating behaviour and related constructs.

In the case of hedonics and food reward, Cheon et al. [186] provide a conceptual model for sweetness hedonics together with measurement implications involving multiple behavioural dimensions. The biological aspects of food hedonics/food reward also yields ideas for conceptualisation and measurement of eating behaviour, possibly with an emphasis on taste, flavour and odour liking [165]. Inhibitory control and regulatory processes together with the contrasting trait of impulsivity [100, 187, 188] have been increasingly emphasized in the conceptualization and measurement of ASR [37] and as being related to weight gain and obesity in children [189–191]. There is an opportunity for scholarship on inhibitory control and impulsivity to contribute further to the conceptualisation and measurement of children’s eating behaviour. It is apparent that there is scope for an increased integration of theory and evidence from these areas into efforts to conceptualise and measure children’s eating behaviour.

### Future Directions

We argued the need for a renewed examination of the core constructs and measures that were included in the body of the present review. This will necessitate attention to theoretical underpinnings, construct definitions and then operationalisation. At the same time, there is a need for the development of an overarching theoretical framework that enables the linking of different eating behaviours and constructs [53]. There are some possible strategies to assist advances in conceptual and theoretical bases of children’s eating behaviours and related constructs.

As an initial step, it is important to recognise that children’s eating behaviour involves the dynamic interaction of multiple behavioural, psychophysiological, psychological, and affective systems in different environments. Conceptualisation and theoretical underpinning should encompass these multiple systems and consider them in context. A comparable “roundtable discussion” to that chaired by Goldsmith [43] on temperament and involving major theorists and approaches to children’s eating behaviour, including emerging approaches, could be helpful in guiding future work on conceptualisation and theoretical underpinnings.

Person-centred analyses of children’s eating behaviours could be helpful. In this approach, instead of treating eating behaviours as single constructs, researchers conceptualize eating behaviours in terms of phenotypes that include patterns or collections of behaviours related to differences in eating behaviours/phenotypes in sub-groups of children. This approach assumes that the person is an integrated totality whose behaviours are interwoven and interacting over time [192, 193]. It is a data driven and exploratory approach [193]. Its contribution at present seems mainly to suggest the presence of different phenotypes that involve a combination or pattern of individual eating behaviours and eating behaviour trajectories. For example, a phenotype of food avoidance trending towards low food approach in infancy, or a phenotype of high and continuing food approach in infancy [194] or a phenotype of dysregulated behaviour (low inhibitory control and high impulsivity) together with higher food approach and lower food avoidance in childhood [102]. The person-centred approach therefore moves the conceptualisation of eating behaviour from individual variables to integrated combinations of variables and trajectories [102, 193, 194]. It places an emphasis on the dynamic interplay between behaviours rather than on individual eating behaviours. We have noted that the contribution of this approach to the conceptualisation of eating behaviour will be enhanced through the development and inclusion of a wider variety and number of individual behaviours included in person-centred analyses [193].

Concept/content mapping could help reduce the conceptual clutter around the theoretical foundations of children’s eating behaviours. Content mapping draws on an expert panel to sort and group relevant constructs together with the relevant terminology and definitions identified from a systematic search [195]. Concept mapping also involves an expert panel in grouping concepts identified from published literature a priori and placing them in named constructs with definitions. Multi-dimensional scaling and hierarchical cluster analysis is then used to arrive at clusters of constructs [196]. Similar strategies with individual eating behaviours could assist in the identification of the main constructs or subdomains of eating behaviour and relationships among them, together with associated definitions.

An additional strategy to increase a focus on the theoretical foundations of children’s eating behaviours could be for journal editors and reviewers to place more emphasis on authors articulating theoretical bases. Finally, efforts at theoretical reviews to identify how theories have been applied and the associated supporting evidence could be helpful [197].

## Conclusions

There still seems to be force in Lumeng & Fisher’s (1) argument that research on children’s eating behaviours should direct more attention to theoretical underpinnings, conceptualisation and measurement. The main eating behaviours that we discussed here seem to have had their origins in relatively practical or applied questions (such as can children “perceive” calories in the case of caloric compensation or whether environmental cues might override children’s sense of fullness in the case of EAH, or how parents perceive their children’s eating in the case of the CEBQ). The measures have served the field well and created a rich body of research. However, the foundation of measurement in science is dependent on the clarity of the theoretical bases and the associated construct definitions. It is in these areas where advances would benefit the field qua science. The implication is that advances in construct definition, operationalisation, and measurement and all that follows (more valid and reliable evidence, clearer targets for intervention and better assessment of intervention outcomes) are dependent on greater regard to the theoretical foundations. Practical outcomes of science, such as contributing to more healthy eating in children, are in turn dependent on the clarity (interpretability) and validity of the associated evidence.

## Data Availability

Not applicable.

## Abbreviations

EAH: Eating in the Absence of Hunger
ASR: Appetite Self-Regulation
DoG: Delay of Gratification
CEBQ: Children’s Eating Behaviour Questionnaire
BEBQ: Baby’s Eating Behaviour Questionnaire
DEBQ-C: Children’s Dutch Eating Behaviour Questionnaire
CTFEQr17: Child and Adolescent Version of the Three-Factor Eating Questionnaire
EC: Effortful Control
EF: Executive Function
BMI: Body Mass Index

## References

[1] Lumeng JC, Fisher JO. Epilogue. In: Lumeng JCF, J. O. , editor. Pediatric Food Preferences and Eating Behaviors. London: Academic Press; 2018. p. 289–92.

[2] Abarca-Gómez L, Abdeen ZA, Hamid ZA, Abu-Rmeileh NM, Acosta-Cazares B, Acuin C (2017). Worldwide trends in body-mass index, underweight, overweight, and obesity from 1975 to 2016: a pooled analysis of 2416 population-based measurement studies in 128·9 million children, adolescents, and adults. The Lancet.

[3] Lumeng JC, Taveras EM, Birch L, Yanovski SZ (2015). Prevention of obesity in infancy and early childhood: a National Institutes of Health workshop. JAMA Pediatr.

[4] Malihi Z, Portch R, Hashemi L, Schlichting D, Wake M, Morton S, et al. Modifiable Early Childhood Risk Factors for Obesity at Age Four Years. Childhood Obesity. 2021;17(3):196-208. 10.1089/chi.2020.0174 33595354

[5] Swinburn BA, Sacks G, Hall KD, McPherson K, Finegood DT, Moodie ML (2011). The global obesity pandemic: shaped by global drivers and local environments. Lancet.

[6] Haboush-Deloye A, Berlin H, Marquez E, Moonie S. Obesity in Early Childhood: Examining the Relationship among Demographic, Behavioral, Nutritional, and Socioeconomic Factors. Child Obes. 2021;17(5):349-56. 10.1089/chi.2020.0263 33944617

[7] Ziauddeen N, Roderick PJ, Macklon NS, Alwan NA (2018). Predicting childhood overweight and obesity using maternal and early life risk factors: a systematic review. Obes Rev.

[8] Zheng M, Lamb KE, Grimes C, Laws R, Bolton K, Ong KK (2018). Rapid weight gain during infancy and subsequent adiposity: a systematic review and meta-analysis of evidence. Obes Res.

[9] Zhou Z, Liew J, Yeh YC, Perez M (2020). Appetitive Traits and Weight in Children: Evidence for Parents' Controlling Feeding Practices as Mediating Mechanisms. J Genet Psychol.

[10] Russell CG, Russell A (2018). Biological and Psychosocial Processes in the Development of Children's Appetitive Traits: Insights from Developmental Theory and Research. Nutrients.

[11] Russell CG, Russell A (2019). A biopsychosocial approach to processes and pathways in the development of overweight and obesity in childhood: Insights from developmental theory and research. Obes Rev.

[12] Harrist AW, Topham GL, Hubbs-Tait L, Page MC, Kennedy TS, Shriver LH. What Developmental Science Can Contribute to a Transdisciplinary Understanding of Childhood Obesity: An Interpersonal and Intrapersonal Risk Model. Child Dev Perspect. 2012;6(4):445-55.

[13] Saltzman JA, Fiese BH, Bost KK, McBride BA (2018). Development of Appetite Self-Regulation: Integrating Perspectives From Attachment and Family Systems Theory. Child Dev Perspect.

[14] Gahagan S (2012). Development of eating behavior: biology and context. J Dev Behav Pediatr.

[15] Harrison K, Bost KK, McBride BA, Donovan SM, Grigsby-Toussaint DS, Kim J (2011). Toward a Developmental Conceptualization of Contributors to Overweight and Obesity in Childhood: The Six-Cs Model. Child Dev Perspect.

[16] Davison KK, Birch LL (2001). Childhood overweight: a contextual model and recommendations for future research. Obes Rev.

[17] Baranowski T, Motil KJ, Moreno JP. Multi-etiological Perspective on Child Obesity Prevention. Curr Nutr Rep. 2019;18(1):1-10. 10.1007/s13668-019-0256-3 PMC6635107 30649714

[18] Bridges LJ, Denham SA, Ganiban JM (2004). Definitional issues in emotion regulation research. Child Dev.

[19] Russell CG, Russell A (2020). "Food" and "non-food" self-regulation in childhood: a review and reciprocal analysis. Int J Behav Nutr Phys Act.

[20] Llewellyn CH, Fildes A (2017). Behavioural Susceptibility Theory: Professor Jane Wardle and the Role of Appetite in Genetic Risk of Obesity. Curr Obes Rep.

[21] Boutelle KN, Manzano MA, Eichen DM (2020). Appetitive traits as targets for weight loss: The role of food cue responsiveness and satiety responsiveness. Physiol Behav.

[22] Carnell S, Benson L, Pryor K, Driggin E (2013). Appetitive traits from infancy to adolescence: using behavioral and neural measures to investigate obesity risk. Physiol Behav.

[23] Kong KL, Anzman-Frasca S, Epstein LH, Eiden RD, Paluch RA (2020). Infants with big appetites: The role of a nonfood environment on infant appetitive traits linked to obesity. Am J Clin Nutr.

[24] Kim SY, Kim YA, Song DY, Bong G, Kim JM, Kim JH (2021). State and Trait Anxiety of Adolescents with Autism Spectrum Disorders. Psychiatry Investig.

[25] Garcia-Garcia I, Neseliler S, Morys F, Dadar M, Yau YHC, Scala SG, et al. Relationship between impulsivity, uncontrolled eating and body mass index: a hierarchical model. Int J Obes. 2021;46(1):129-36. 10.1038/s41366-021-00966-4 34552208

[26] Guerrieri R, Nederkoorn C, Stankiewicz K, Alberts H, Geschwind N, Martijn C (2007). The influence of trait and induced state impulsivity on food intake in normal-weight healthy women. Appetite.

[27] Papaioannou MA, Micheli N, Power TG, Fisher JO, Hughes SO (2021). Associations Between Independent Assessments of Child Appetite Self-Regulation: A Narrative Review. Front Nutr.

[28] Hohman EE, McNitt KM, Eagleton SG, Francis LA, Keller KL, Savage JS (2021). Validation of a Classroom Version of the Eating in the Absence of Hunger Paradigm in Preschoolers. Front Nutr.

[29] Kan C, Herle M, Treasure J, Jones A, Rijsdijk F, Llewellyn C (2020). Common etiological architecture underlying reward responsiveness, externally driven eating behaviors, and BMI in childhood: findings from the Gemini twin cohort. Int J Obes.

[30] Llewellyn CH, van Jaarsveld CH, Plomin R, Fisher A, Wardle J (2012). Inherited behavioral susceptibility to adiposity in infancy: a multivariate genetic analysis of appetite and weight in the Gemini birth cohort. Am J Clin Nutr.

[31] Papaioannou MA, Micheli N, Power TG, O'Connor TM, Fisher JO, Hughes SO. Maternal Feeding Styles and Child Appetitive Traits: Direction of Effects in Hispanic Families With Low Incomes. Frontiers in Public Health. 2022;10. Article 871923. 10.3389/fpubh.2022.871923 PMC9201210 35719648

[32] Carnell S, Wardle J (2009). Appetitive traits in children. New evidence for associations with weight and a common, obesity-associated genetic variant. Appetite.

[33] French SA, Epstein LH, Jeffery RW, Blundell JE, Wardle J (2012). Eating behavior dimensions. Associations with energy intake and body weight. A review. Appetite.

[34] Kininmonth A, Smith A, Carnell S, Steinsbekk S, Fildes A, Llewellyn C. The association between childhood adiposity and appetite assessed using the Child Eating Behavior Questionnaire and Baby Eating Behavior Questionnaire: A systematic review and meta-analysis. Obesity reviews : an official journal of the International Association for the Study of Obesity. 2021;22(5):e13169. 10.1111/obr.13169 33554425

[35] Kral TVE, Moore RH, Chittams J, Jones E, O'Malley L, Fisher JO (2018). Identifying behavioral phenotypes for childhood obesity. Appetite.

[36] Lee J, Keast R, Russell CG. The biological foundations of children’s food fussiness: Systematic review with narrative synthesis. Food Quality and Preference. 2021;97. Article 104477.

[37] Russell A, Russell CG (2021). Appetite self-regulation declines across childhood while general self-regulation improves: A narrative review of the origins and development of appetite self-regulation. Appetite.

[38] Taylor CM, Wernimont SM, Northstone K, Emmett PM (2015). Picky/fussy eating in children: Review of definitions, assessment, prevalence and dietary intakes. Appetite.

[39] Brownell CA, Lemerise EA, Pelphrey KA, Roisman GI. Measuring Socioemotional Development. Handb Child Psychol Dev Sci. 2015;3:1–46.

[40] Flake JK, Fried EI (2020). Measurement Schmeasurement: Questionable Measurement Practices and How to Avoid Them. Adv Methods Pract Psychol Sci.

[41] Rothbart MK, Ahadi SA, Hershey KL, Fisher P (2001). Investigations of temperament at three to seven years: The Children's Behavior Questionnaire. Child Dev.

[42] Rubin KH, Chronis-Tuscano A (2021). Perspectives on Social Withdrawal in Childhood: Past, Present, and Prospects. Child Dev Perspect.

[43] Goldsmith HH, Buss AH, Plomin R, Rothbart MK, Thomas A, Chess S (1987). Roundtable: What Is Temperament? Four Approaches. Child Dev.

[44] Shiner RL, DeYoung, C. G. The structure of temperament and personality traits: A developmental perspective. In: Zelazo PD, editor. The Oxford handbook of developmental psychology, Vol 2 Self and other (pp 113–141). 2. Oxford: Oxford University Press; 2015.

[45] Kagan J, Reznick JS, Snidman N (1987). The physiology and psychology of behavioral inhibition in children. Child Dev.

[46] Kagan J, Reznick JS, Snidman N, Gibbons J, Johnson MO (1988). Childhood derivatives of inhibition and lack of inhibition to the unfamiliar. Child Dev.

[47] Goldsmith HH, Rieser-Danner LA, Briggs S (1991). Evaluating convergent and discriminant validity of temperament questionnaires for preschoolers, toddlers, and infants. Dev Psychol.

[48] Bates JE, Pettit GS, Grusec JE, Hastings PD (2015). Temperament, Parenting, and Social Development. Handbook of Socialization: Theory and Research.

[49] Bornstein MH, Putnick DL, Gartstein MA, Hahn CS, Auestad N, O'Connor DL (2015). Infant temperament: stability by age, gender, birth order, term status, and socioeconomic status. Child Dev.

[50] Moding KJ, Stifter CA (2016). Temperamental approach/withdrawal and food neophobia in early childhood: Concurrent and longitudinal associations. Appetite.

[51] Inzlicht M, Werner KM, Briskin JL, Roberts BW (2021). Integrating Models of Self-Regulation. Annu Rev Psychol.

[52] Darling-Churchill KE, Lippman L (2016). Early childhood social and emotional development: Advancing the field of measurement. J Appl Dev Psychol.

[53] Muthukrishna M, Henrich J (2019). A problem in theory. Nat Hum Behav.

[54] Overton WF (2007). A Coherent Metatheory for Dynamic Systems: Relational Organicism-Contextualism. Hum Dev.

[55] Witherington DC (2007). The Dynamic Systems Approach as Metatheory for Developmental Psychology. Hum Dev.

[56] Keller KL, Kling SMR, Fuchs B, Pearce AL, Reigh NA, Masterson T (2019). A Biopsychosocial Model of Sex Differences in Children's Eating Behaviors. Nutrients.

[57] Chawner LR, Hetherington MM (2021). Utilising an integrated approach to developing liking for and consumption of vegetables in children. Physiol Behav.

[58] Liew J, Zhou Z, Perez M, Yoon M, Kim M (2020). Parental Child-feeding in the Context of Child Temperament and Appetitive Traits: Evidence for a Biopsychosocial Process Model of Appetite Self-Regulation and Weight Status. Nutrients.

[59] Jones SM, Zaslow M, Darling-Churchill KE, Halle TG (2016). Assessing early childhood social and emotional development: Key conceptual and measurement issues. J Appl Dev Psychol.

[60] Wardle J, Guthrie CA, Sanderson S, Rapoport L (2001). Development of the Children's Eating Behaviour Questionnaire. J Child Psychol Psychiatry.

[61] Llewellyn CH, van Jaarsveld CH, Johnson L, Carnell S, Wardle J (2011). Development and factor structure of the Baby Eating Behaviour Questionnaire in the Gemini birth cohort. Appetite.

[62] van Strien T, Oosterveld P (2008). The children's DEBQ for assessment of restrained, emotional, and external eating in 7- to 12-year-old children. Int J Eat Disord.

[63] van Strien T, Frijters JER, Bergers GPA, Defares PB (1986). The Dutch Eating Behavior Questionnaire (DEBQ) for assessment of restrained, emotional, and external eating behavior. Int J Eat Disord.

[64] Bryant EJ, Thivel D, Chaput JP, Drapeau V, Blundell JE, King NA (2018). Development and validation of the Child Three-Factor Eating Questionnaire (CTFEQr17). Public Health Nutr.

[65] Tan CC, Holub SC (2011). Children's self-regulation in eating: associations with inhibitory control and parents' feeding behavior. J Pediatr Psychol.

[66] Faith MS, Johnson SL, Allison DB (1997). Putting the behavior into the behavior genetics of obesity. Behav Genet.

[67] Eagleton SG, Na M, Savage JS (2022). Food insecurity is associated with higher food responsiveness in low-income children: The moderating role of parent stress and family functioning. Pediatr Obes.

[68] Russell CG, Worsley T (2016). Associations between appetitive traits and food preferences in preschool children. Food Qual Prefer.

[69] Domoff SE, Miller AL, Kaciroti N, Lumeng JC (2015). Validation of the Children's Eating Behaviour Questionnaire in a low-income preschool-aged sample in the United States. Appetite.

[70] Ayre S, Gallegos D, Nambiar S, Tran CQ, Do DN, Jansen E. Preliminary exploration of the use of the Children's Eating Behaviour Questionnaire (CEBQ) and Feeding Practices and Structure Questionnaire (FPSQ) in Vietnamese mothers. Eur J Clin Nutr. 2021;76(3):442-9. 10.1038/s41430-021-00947-w 34302134

[71] Oyama S, Arslanian KJ, Fidow UT, Naseri T, Soti-Ulberg C, Hawley NL (2021). Factorial Validation Analysis of the Baby and Children’s Eating Behavior Questionnaires in Samoa. Eat Behav.

[72] Quah PL, Chan YH, Aris IM, Pang WW, Toh JY, Tint MT (2015). Prospective associations of appetitive traits at 3 and 12 months of age with body mass index and weight gain in the first 2 years of life. BMC Pediatr.

[73] Sleddens EF, Kremers SP, Thijs C (2008). The children's eating behaviour questionnaire: factorial validity and association with Body Mass Index in Dutch children aged 6–7. Int J Behav Nutr Phys Act.

[74] Svensson V, Lundborg L, Cao Y, Nowicka P, Marcus C, Sobko T (2011). Obesity related eating behaviour patterns in Swedish preschool children and association with age, gender, relative weight and parental weight - factorial validation of the Children's Eating Behaviour Questionnaire. Int J Behav Nutr Phys Act.

[75] Cao Y-T, Svensson V, Marcus C, Zhang J, Zhang J-D, Sobko T (2012). Eating behaviour patterns in Chinese children aged 12–18 months and association with relative weight - factorial validation of the Children's Eating Behaviour Questionnaire. Int J Behav Nutr Phys Act.

[76] Santos JL, Ho-Urriola JA, González A, Smalley SV, Domínguez-Vásquez P, Cataldo R (2011). Association between eating behavior scores and obesity in Chilean children. Nutr J.

[77] Sparks MA, Radnitz CL (2012). Confirmatory factor analysis of the Children's Eating Behaviour Questionnaire in a low-income sample. Eat Behav.

[78] Kagan J (2022). Temperamental and Theoretical Contributions to Clinical Psychology. Annu Rev Clin Psychol.

[79] Brown A, Lee M (2012). Breastfeeding during the first year promotes satiety responsiveness in children aged 18–24 months. Pediatr Obes.

[80] Burgess B, Faith MS. Satiety Responsiveness and Eating Rate in Childhood: Development, Plasticity, and the Family Footprint. In: Lumeng JCF, J., editor. Pediatric Food Preferences and Eating Behaviors. London: Academic Press; 2018. p. 93–110.

[81] Russell CG, Denney-Wilson E, Laws RA, Abbott G, Zheng M, Lymer SJ (2018). Impact of the growing healthy mHealth program on maternal feeding practices, infant food preferences, and satiety responsiveness: quasi-experimental study. JMIR Mhealth Uhealth.

[82] Forde CG, Ares G, Varela PA (2018). Measuring satiation and satiety. Woodhead Publishing Series in Food Science, Technology and Nutrition.

[83] Ek A, Sorjonen K, Eli K, Lindberg L, Nyman J, Marcus C (2016). Associations between Parental Concerns about Preschoolers' Weight and Eating and Parental Feeding Practices: Results from Analyses of the Child Eating Behavior Questionnaire, the Child Feeding Questionnaire, and the Lifestyle Behavior Checklist. PLoS ONE.

[84] Hughes SO, Frazier-Wood AC (2016). Satiety and the Self-Regulation of Food Take in Children: a Potential Role for Gene-Environment Interplay. Curr Obes Rep.

[85] Miller AL, Gearhardt AN, Fredericks EM, Katz B, Shapiro LF, Holden K (2018). Targeting self-regulation to promote health behaviors in children. Behav Res Ther.

[86] Hughes SO, Power TG, O'Connor TM, Orlet FJ (2015). Executive functioning, emotion regulation, eating self-regulation, and weight status in low-income preschool children: how do they relate?. Appetite.

[87] Bellisle F, Drewnowski A, Anderson GH, Westerterp-Plantenga M, Martin CK (2012). Sweetness, satiation, and satiety. J Nutr.

[88] Frankel LA, O'Connor TM, Chen TA, Nicklas T, Power TG, Hughes SO (2014). Parents' perceptions of preschool children's ability to regulate eating Feeding style differences. Appetite.

[89] Monnery-Patris S, Rigal N, Peteuil A, Chabanet C, Issanchou S (2019). Development of a new questionnaire to assess the links between children's self-regulation of eating and related parental feeding practices. Appetite.

[90] Sherry B, McDivitt J, Birch LL, Cook FH, Sanders S, Prish JL (2004). Attitudes, practices, and concerns about child feeding and child weight status among socioeconomically diverse white, Hispanic, and African-American mothers. J Am Diet Assoc.

[91] Nigg JT (2017). Annual Research Review: On the relations among self-regulation, self-control, executive functioning, effortful control, cognitive control, impulsivity, risk-taking, and inhibition for developmental psychopathology. J Child Psychol Psychiatry.

[92] Bridgett DJ, Burt NM, Edwards ES, Deater-Deckard K (2015). Intergenerational transmission of self-regulation: A multidisciplinary review and integrative conceptual framework. Psychol Bull.

[93] Van Malderen E, Goossens L, Verbeken S, Kemps E (2020). Multi-method evidence for a dual-pathway perspective of self-regulation in loss of control over eating among adolescents. Appetite.

[94] Reigh NA, Rolls BJ, Francis LA, Buss KA, Hayes JE, Hetherington MM (2022). Examining the Role of Food Form on Children's Self-Regulation of Energy Intake. Front Nutr.

[95] Berthoud HR, Munzberg H, Morrison CD (2017). Blaming the Brain for Obesity: Integration of Hedonic and Homeostatic Mechanisms. Gastroenterology.

[96] Lowe MR, Butryn ML (2007). Hedonic hunger: a new dimension of appetite?. Physiol Behav.

[97] Mennella JA, Bobowski NK, Reed DR (2016). The development of sweet taste: From biology to hedonics. Rev Endocr Metab Disord.

[98] Smith AD, Herle M, Fildes A, Cooke L, Steinsbekk S, Llewellyn CH (2017). Food fussiness and food neophobia share a common etiology in early childhood. J Child Psychol Psychiatry.

[99] Stoeckel LE, Birch LL, Heatherton T, Mann T, Hunter C, Czajkowski S (2017). Psychological and neural contributions to appetite self-regulation. Obesity (Silver Spring).

[100] Lelakowska G, Kanya MJ, Balassone BR, Savoree SL, Boddy LE, Power TG (2019). Toddlers' impulsivity, inhibitory control, and maternal eating-related supervision in relation to toddler body mass index: Direct and interactive effects. Appetite.

[101] Gearhardt AN. Role of Reward Pathways in Appetitive Drive and Regulation. In: Lumeng JCF, J., editor. Pediatric Food Preferences and Eating Behaviors. London: Academic Press; 2018. p. 111–26.

[102] Francis LA, Rollins BY, Keller KL, Nix RL, Savage JS (2022). Profiles of Behavioral Self-Regulation and Appetitive Traits in Preschool Children: Associations With BMI and Food Parenting Practices. Front Nutr.

[103] Blair C, Razza RP (2007). Relating effortful control, executive function, and false belief understanding to emerging math and literacy ability in kindergarten. Child Dev.

[104] Verdejo-Garcia A, Tiego J, Kakoschke N, Moskovsky N, Voigt K, Anderson A (2021). A unified online test battery for cognitive impulsivity reveals relationships with real-world impulsive behaviours. Nat Hum Behav.

[105] Tiego J, Testa R, Bellgrove MA, Pantelis C, Whittle S (2018). A Hierarchical Model of Inhibitory Control. Front Psychol.

[106] Thamotharan S, Lange K, Zale EL, Huffhines L, Fields S (2013). The role of impulsivity in pediatric obesity and weight status: a meta-analytic review. Clin Psychol Rev.

[107] Simpson A, Carroll DJ (2019). Understanding Early Inhibitory Development: Distinguishing Two Ways That Children Use Inhibitory Control. Child Dev.

[108] Hendry A, Greenhalgh I, Bailey R, Fiske A, Dvergsdal H, Holmboe K (2021). Development of directed global inhibition, competitive inhibition and behavioural inhibition during the transition between infancy and toddlerhood. Dev Sci.

[109] Bennett C, Blissett J (2019). Multiple measures of impulsivity, eating behaviours and adiposity in 7–11-year-olds. Appetite.

[110] Gagne JR, Liew J, Nwadinobi OK. “How does the broader construct of self-regulation relate to emotion regulation in young children?”. Developmental Review. 2021;60. Article 100965.

[111] Saltzman JA, Fiese BH, Bost KK, McBride BA (2017). Development of Appetite Self-Regulation: Integrating Perspectives From Attachment and Family Systems Theory. Child Dev Perspect.

[112] Giuliani NR, Kelly NR (2022). Associations Among Food Delay of Gratification, Cognitive Measures, and Environment in a Community Preschool Sample. Front Nutr.

[113] Seeyave DM, Coleman S, Appugliese D, Corwyn RF, Bradley RH, Davidson NS (2009). Ability to delay gratification at age 4 years and risk of overweight at age 11 years. Arch Pediatr Adolesc Med.

[114] Lundquist E, Austen M, Bermudez M, Rubin C, Bruce AS, Masterson TD (2019). Time spent looking at food during a delay of gratification task is positively associated with children's consumption at ad libitum laboratory meals. Appetite.

[115] Mischel W, Ebbesen EB (1970). Attention in delay of gratification. J Pers Soc Psychol.

[116] Mischel W, Ebbesen EB, Zeiss AR (1972). Cognitive and attentional mechanisms in delay of gratification. J Pers Soc Psychol.

[117] Mischel W, Shoda Y, Rodriguez MI (1989). Delay of gratification in children. Science.

[118] Duran CAK, Grissmer DW (2020). Choosing immediate over delayed gratification correlates with better school-related outcomes in a sample of children of color from low-income families. Dev Psychol.

[119] Hongwanishkul D, Happaney KR, Lee WS, Zelazo PD (2005). Assessment of hot and cool executive function in young children: age-related changes and individual differences. Dev Neuropsychol.

[120] Watts TW, Duncan GJ, Quan H (2018). Revisiting the Marshmallow Test: A Conceptual Replication Investigating Links Between Early Delay of Gratification and Later Outcomes. Psychol Sci.

[121] Watts TW, Duncan GJ (2020). Controlling, Confounding, and Construct Clarity: Responding to Criticisms of "Revisiting the Marshmallow Test" by Doebel, Michaelson, and Munakata (2020) and Falk, Kosse, and Pinger (2020). Psychol Sci.

[122] Suor JH, Sturge-Apple ML, Jones-Gordils HR (2019). Parsing profiles of temperamental reactivity and differential routes to delay of gratification: A person-based approach. Dev Psychopathol.

[123] Birch LL, Fisher JO (2000). Mothers' child-feeding practices influence daughters' eating and weight. Am J Clin Nutr.

[124] Fisher JO, Birch LL (1999). Restricting access to foods and children's eating. Appetite.

[125] Fisher JO, Birch LL (2000). Parents’ Restrictive Feeding Practices are Associated with Young Girls’ Negative Self-evaluation of Eating. J Am Diet Assoc.

[126] Fisher JO, Birch LL (2002). Eating in the absence of hunger and overweight in girls from 5 to 7 y of age. Am J Clin Nutr.

[127] Boone-Heinonen J, Weeks HM, Sturza J, Miller AL, Lumeng JC, Bauer KW (2019). Prenatal predictors of objectively measured appetite regulation in low-income toddlers and preschool-age children. Pediatr Obes.

[128] Stojek MMK, MacKillop J (2017). Relative reinforcing value of food and delayed reward discounting in obesity and disordered eating: A systematic review. Clin Psychol Rev.

[129] Shapiro ALB, Johnson SL, Sutton B, Legget KT, Dabelea D, Tregellas JR (2019). Eating in the absence of hunger in young children is related to brain reward network hyperactivity and reduced functional connectivity in executive control networks. Pediatr Obes.

[130] Miller AL, Gearhardt AN, Retzloff L, Sturza J, Kaciroti N, Lumeng JC (2018). Early Childhood Stress and Child Age Predict Longitudinal Increases in Obesogenic Eating Among Low-Income Children. Acad Pediatr.

[131] Hill C, Llewellyn CH, Saxton J, Webber L, Semmler C, Carnell S (2008). Adiposity and'eating in the absence of hunger'in children. Int J Obes.

[132] Francis LA, Riggs NR. Executive Function and Self-Regulatory Influences on Children's Eating. In: Lumeng JCF, J., editor. Pediatric Food Preferences and Eating Behaviors. London: Academic Press; 2018. p. 183–206.

[133] Francis LA, Ventura AK, Marini M, Birch LL (2007). Parent overweight predicts daughers’ increase in BMI and disinhibited overeating from 5 to 13 years. Obesity.

[134] Carnell S, Wardle J (2007). Measuring behavioural susceptibility to obesity: validation of the child eating behaviour questionnaire. Appetite.

[135] Jackson R, Haszard JJ, Morrison S, Galland BC, McIntosh D, Ward AL (2021). Measuring short-term eating behaviour and desire to eat: Validation of the child eating behaviour questionnaire and a computerized 'desire to eat' computerized questionnaire. Appetite.

[136] Shomaker LB, Tanofsky-Kraff M, Yanovski JA. Disinhibited Eating and Body Weight in Youth. In: Preedy VR, Watson RR, Martin CR, editors. Handbook of Behavior, Food and Nutrition. New York, NY: Springer New York; 2011. p. 2183–200.

[137] Madowitz J, Liang J, Peterson CB, Rydell S, Zucker NL, Tanofsky-Kraff M (2014). Concurrent and convergent validity of the eating in the absence of hunger questionnaire and behavioral paradigm in overweight children. Int J Eat Disord.

[138] Kral TV, Allison DB, Birch LL, Stallings VA, Moore RH, Faith MS (2012). Caloric compensation and eating in the absence of hunger in 5- to 12-y-old weight-discordant siblings. Am J Clin Nutr.

[139] Kral TVE, Moore RH, Chittams J, O'Malley L, Jones E, Quinn RJ (2020). Does eating in the absence of hunger extend to healthy snacks in children?. Pediatr Obes.

[140] Miller AL, Riley H, Domoff SE, Gearhardt AN, Sturza J, Kaciroti N (2019). Weight status moderates stress-eating in the absence of hunger associations in children. Appetite.

[141] Giuliani NR, Kelly NR (2021). Delay of Gratification Predicts Eating in the Absence of Hunger in Preschool-Aged Children. Front Psychol.

[142] Fogel A, McCrickerd K, Goh AT, Fries LR, Chong YS, Tan KH (2019). Associations between inhibitory control, eating behaviours and adiposity in 6-year-old children. Int J Obes.

[143] Birch LL, Deysher M (1985). Conditioned and unconditioned caloric compensation: Evidence for self-regulation of food intake in young children. Learn Motiv.

[144] Rolls BJ (2009). The relationship between dietary energy density and energy intake. Physiol Behav.

[145] Rolls BJ, Kling SMR, Keller KL, Zuraikat FM, Sanchez CE, Roe LS (2019). Portion size has sustained effects over 5 days in preschool children: a randomized trial. Am J Clin Nutr.

[146] Almiron-Roig E, Palla L, Guest K, Ricchiuti C, Vint N, Jebb SA (2013). Factors that determine energy compensation: a systematic review of preload studies. Nutr Rev.

[147] Carnell S, Benson L, Gibson EL, Mais LA, Warkentin S (2017). Caloric compensation in preschool children: Relationships with body mass and differences by food category. Appetite.

[148] Kral TV, Stunkard AJ, Berkowitz RI, Stallings VA, Brown DD, Faith MS (2007). Daily food intake in relation to dietary energy density in the free-living environment: a prospective analysis of children born at different risk of obesity. Am J Clin Nutr.

[149] Remy E, Issanchou S, Chabanet C, Boggio V, Nicklaus S (2015). Impact of adiposity, age, sex and maternal feeding practices on eating in the absence of hunger and caloric compensation in preschool children. Int J Obes.

[150] Gibbons C, Hopkins M, Beaulieu K, Oustric P, Blundell JE (2019). Issues in Measuring and Interpreting Human Appetite (Satiety/Satiation) and Its Contribution to Obesity. Curr Obes Rep.

[151] Cooke L, Wardle J, Gibson EL (2003). Relationship between parental report of food neophobia and everyday food consumption in 2–6-year-old children. Appetite.

[152] Dovey TM, Staples PA, Gibson EL, Halford JC (2008). Food neophobia and 'picky/fussy' eating in children: a review. Appetite.

[153] Brown CL, Vander Schaaf EB, Cohen GM, Irby MB, Skelton JA (2016). Association of Picky Eating and Food Neophobia with Weight: A Systematic Review. Child Obes.

[154] Cole NC, An R, Lee SY, Donovan SM (2017). Correlates of picky eating and food neophobia in young children: a systematic review and meta-analysis. Nutr Rev.

[155] Rioux C, Lafraire J, Picard D (2017). The Child Food Rejection Scale: Development and validation of a new scale to assess food neophobia and pickiness among 2- to 7-year-old French children. Eur Rev Appl Psychol.

[156] Moding KJ, Stifter CA (2016). Stability of food neophobia from infancy through early childhood. Appetite.

[157] Rioux C, Lafraire J, Picard D (2017). The child food rejection scale: development and validation of a new scale to assess food neophobia and pickiness among 2-to 7-year-old French children. Eur Rev Appl Psychol.

[158] Anzman‐Frasca S, Moding KJ, Forestell CA, Francis LA. Applying developmental science concepts to improve the applicability of children’s food preference learning research. Child Dev Perspect. 2022;16(3):180-7.

[159] Gottlieb G, Wahlsten D, Lickliter R, Lerner RM, Damon W (2007). The Significance of Biology for Human Development: A Developmental Psychobiological Systems View. Handbook of Child Psychology.

[160] Lowe CJ, Morton JB, Reichelt AC (2020). Adolescent obesity and dietary decision making-a brain-health perspective. Lancet Child Adolesc Health.

[161] Lowe CJ, Reichelt AC, Hall PA (2019). The Prefrontal Cortex and Obesity: A Health Neuroscience Perspective. Trends Cogn Sci.

[162] Erickson KI, Creswell JD, Verstynen TD, Gianaros PJ (2014). Health Neuroscience: Defining a New Field. Curr Dir Psychol Sci.

[163] Epstein LH, Carr KA (2021). Food reinforcement and habituation to food are processes related to initiation and cessation of eating. Physiol Behav.

[164] Higgs S, Spetter MS, Thomas JM, Rotshtein P, Lee M, Hallschmid M (2017). Interactions between metabolic, reward and cognitive processes in appetite control: Implications for novel weight management therapies. J Psychopharmacol.

[165] de Araujo IE, Schatzker M, Small DM (2020). Rethinking Food Reward. Annu Rev Psychol.

[166] Freitas A, Albuquerque G, Silva C, Oliveira A (2018). Appetite-Related Eating Behaviours: An Overview of Assessment Methods, Determinants and Effects on Children's Weight. Ann Nutr Metab.

[167] Ziauddeen H, Alonso-Alonso M, Hill JO, Kelley M, Khan NA (2015). Obesity and the neurocognitive basis of food reward and the control of intake. Adv Nutr.

[168] Morales I, Berridge KC (2020). 'Liking' and 'wanting' in eating and food reward: Brain mechanisms and clinical implications. Physiol Behav.

[169] Ha OR, Lim SL, Bruce AS (2020). Neural Mechanisms of Food Decision-Making in Children. Curr Nutr Rep.

[170] Alonso-Alonso M, Woods SC, Pelchat M, Grigson PS, Stice E, Farooqi S (2015). Food reward system: current perspectives and future research needs. Nutr Rev.

[171] Tiego J, Bellgrove MA, Whittle S, Pantelis C, Testa R (2020). Common mechanisms of executive attention underlie executive function and effortful control in children. Dev Sci.

[172] Egbert AH, Creber C, Loren DM, Bohnert AM (2019). Executive function and dietary intake in youth: A systematic review of the literature. Appetite.

[173] Gagne JR (2017). Self-Control in Childhood: A Synthesis of Perspectives and Focus on Early Development. Child Dev Perspect.

[174] Giuliani NR, Merchant JS, Cosme D, Berkman ET (2018). Neural predictors of eating behavior and dietary change. Ann N Y Acad Sci.

[175] Hayes JF, Eichen DM, Barch DM, Wilfley DE (2018). Executive function in childhood obesity: Promising intervention strategies to optimize treatment outcomes. Appetite.

[176] Keller KL, Bruce AS. Neurocognitive Influences on Eating Behavior in Children. In: Lumeng JCF, J., editor. Pediatric Food Preferences and Eating Behaviors. London: Academic Press; 2018. p. 207–31.

[177] Rhee KE, Manzano M, Goffin S, Strong D, Boutelle KN (2021). Exploring the relationship between appetitive behaviours, executive function, and weight status among preschool children. Pediatr Obes..

[178] Doom JR, Young ES, Farrell AK, Roisman GI, Simpson JA. Behavioral, cognitive, and socioemotional pathways from early childhood adversity to BMI: Evidence from two prospective, longitudinal studies. Dev Psychopathol. 2022:1–17. 10.1017/S0954579421001887 PMC9652481 35545317

[179] Blundell J, Rogers, PJ., & Hill, AJ. . Evaluating the satiating power of foods: implications for acceptance and consumption. . In: J. Colms DB, RM. Pangborn, & O. Raunhardt editor. Food acceptance and nutrition London: Academic Press; 1987. p. 205–19.

[180] Amin T, Mercer JG (2016). Hunger and Satiety Mechanisms and Their Potential Exploitation in the Regulation of Food Intake. Curr Obes Rep.

[181] Blundell J, de Graaf C, Hulshof T, Jebb S, Livingstone B, Lluch A (2010). Appetite control: methodological aspects of the evaluation of foods. Obes Rev.

[182] Chambers L (2016). Food texture and the satiety cascade. Nutr Bull.

[183] de Graaf C, Blom WA, Smeets PA, Stafleu A, Hendriks HF (2004). Biomarkers of satiation and satiety. Am J Clin Nutr.

[184] Benelam B (2009). Satiation, satiety and their effects on eating behaviour. Nutr Bull.

[185] Casanova N, Finlayson G, Blundell JE, Hopkins M (2019). Biopsychology of human appetite — understanding the excitatory and inhibitory mechanisms of homeostatic control. Curr Opin Physio.

[186] Cheon E, Reister EJ, Hunter SR, Mattes RD (2021). Finding the Sweet Spot: Measurement, Modification, and Application of Sweet Hedonics in Humans. Adv Nutr..

[187] Bennett C, Blissett J (2020). Interactive effects of impulsivity and dietary restraint over snack intake in children. Appetite.

[188] Jasinska AJ, Yasuda M, Burant CF, Gregor N, Khatri S, Sweet M (2012). Impulsivity and inhibitory control deficits are associated with unhealthy eating in young adults. Appetite.

[189] Houben K, Nederkoorn C, Jansen A (2014). Eating on impulse: the relation between overweight and food-specific inhibitory control. Obesity (Silver Spring).

[190] Nederkoorn C, Braet C, Van Eijs Y, Tanghe A, Jansen A (2006). Why obese children cannot resist food: the role of impulsivity. Eat Behav.

[191] Kemps E, Goossens L, Petersen J, Verbeken S, Vervoort L, Braet C (2020). Evidence for enhancing childhood obesity treatment from a dual-process perspective: A systematic literature review. Clin Psychol Rev.

[192] Bergman LR, Trost K (2006). The Person-Oriented Versus the Variable-Oriented Approach: Are They Complementary, Opposites, or Exploring Different Worlds?. Merrill Palmer Quart.

[193] Russell A, Leech RM, Russell CG (2021). Conceptualizing and Measuring Appetite Self-Regulation Phenotypes and Trajectories in Childhood: A Review of Person-Centered Strategies. Front Nutr.

[194] Russell CG, Appleton J, Burnett AJ, Rossiter C, Fowler C, Denney-Wilson E (2021). Infant Appetitive Phenotypes: A Group-Based Multi-Trajectory Analysis. Front Nutr.

[195] Vaughn AE, Ward DS, Fisher JO, Faith MS, Hughes SO, Kremers SP (2016). Fundamental constructs in food parenting practices: a content map to guide future research. Nutr Rev.

[196] O'Connor TM, Masse LC, Tu AW, Watts AW, Hughes SO, Beauchamp MR (2017). Food parenting practices for 5 to 12 year old children: a concept map analysis of parenting and nutrition experts input. Int J Behav Nutr Phys Act.

[197] Turner JR, Baker R, Kellner F (2018). Theoretical Literature Review: Tracing the Life Cycle of a Theory and Its Verified and Falsified Statements. Hum Resour Dev Rev.

